# La migration d'un cathéter de dérivation ventriculo-péritonéale

**DOI:** 10.11604/pamj.2014.17.21.2248

**Published:** 2014-01-17

**Authors:** Hassan Baallal, Brahim El Mostarchid

**Affiliations:** 1 Department of Neurosurgery, Mohammed V Military Teaching Hospital, University of King Mohammed V Souissi, Rabat, Morocco

**Keywords:** Cathéter de dérivation, hydrocéphalie, liquide cérébrospinal, Shunt catheter, hydrocephalus, cerebrospinal fluid

## Image en medicine

La connaissance de plus en plus précise des mécanismes physiopathologiques des hydrocéphalies a conduit aux progrès actuels dans la conception des shunts. La maîtrise des techniques chirurgicales de dérivations liquidiennes a totalement modifié leur pronostic. Cependant, qu'elle soit péritonéale ou atriale la dérivation du liquide cérébrospinal (LCS) n'est pas un geste anodin puisque plusieurs types de complication ont été décrits. Nous rapportons une migration de l'extrémité proximale du cathéter de dérivation ventriculo-péritonéale dans la cavité ventriculaire comme montre cette image scanographique.

**Figure 1:**
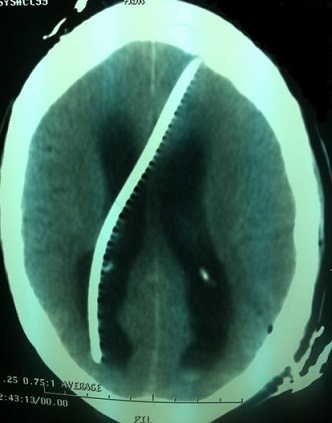
Coupe axiale d'une TDM cérébrale montrant La migration d'un cathéter de dérivation ventriculo-péritonéale

